# Work-Related Health Disorders among Saudi Computer Users

**DOI:** 10.1155/2014/723280

**Published:** 2014-10-14

**Authors:** Ibrahim M. Jomoah

**Affiliations:** Department of Industrial Engineering, Faculty of Engineering, King Abdulaziz University (KAU), P.O. Box 80204, Jeddah 21589, Saudi Arabia

## Abstract

The present study was conducted to investigate the prevalence of musculoskeletal disorders and eye and vision complaints among the computer users of King Abdulaziz University (KAU), Saudi Arabian Airlines (SAUDIA), and Saudi Telecom Company (STC). Stratified random samples of the work stations and operators at each of the studied institutions were selected and the ergonomics of the work stations were assessed and the operators' health complaints were investigated. The average ergonomic score of the studied work station at STC, KAU, and SAUDIA was 81.5%, 73.3%, and 70.3, respectively. Most of the examined operators use computers daily for ≤ 7 hours, yet they had some average incidences of general complaints (e.g., headache, body fatigue, and lack of concentration) and relatively high level of incidences of eye and vision complaints and musculoskeletal complaints. The incidences of the complaints have been found to increase with the (a) decrease in work station ergonomic score, (b) progress of age and duration of employment, (c) smoking, (d) use of computers, (e) lack of work satisfaction, and (f) history of operators' previous ailments. It has been recommended to improve the ergonomics of the work stations, set up training programs, and conduct preplacement and periodical examinations for operators.

## 1. Introduction

The one thing that has had the greatest impact on our lives in modern time is the computer. Along with smaller size and affordable prices, there has been the advent of the Internet. This has ensured that people use this technology either at their work place or at home. Meanwhile, the applications of computer technology and the accompanying use of video display terminals (VDTs) are revolutionizing the workplaces worldwide, and their use will continue to grow in the future.

Although these developments may perform operators' tasks efficiently, they could face some factors such as work stress, repetitious tasks, boredom, interpersonal factors, unsafe postures, and poor design of workstation that will negatively affect their health, performance, and productivity. For example, the development of VDTs technology may have contributed to the increase of users' health problems such as cumulative trauma disorders (CTDs) of upper extremity and back pain [1–54] as well as vision problems [1–11, 13, 14, 19, 20, 26, 44, 45, 51–53, 55–84].

However, the application of ergonomics principles to office workstations will reduce such health risks. For example, one of the goals of the ergonomic processes is to design or modify people's work and other activities to be within their capabilities and limitations [3, 5–7, 12, 15–17, 22, 23, 28–30, 38, 44–46, 85–88]. One possible outcome of poor harmonization is disorder of the musculoskeletal system known as repetitive strain injuries (RSI), CTD, or activity and work-related musculoskeletal disorder (WMSD). Those working in office-type jobs involving keyboarding and other computer related activities suffer from these disorders [9, 13, 15–18, 22–24, 28, 33, 42, 50, 88].

Currently computer related injuries are developing into an epidemic among computer users. It is estimated that, worldwide, 25% of computer users are already suffering from computer related injuries [35]. The United States has to shell out more than 2 billion US dollars annually for having ignored these computer related problems. It is now proved that the duration of work and computer-related problems are positively correlated. It is not uncommon these days for people having to leave computer dependent careers or even be permanently disabled and unable to perform tasks such as driving or dressing themselves. Occupationally caused RSI rank first among the health problems potentially affecting the quality of life [89]. Meanwhile, poor workstation design and poor ergonomics have been associated with an increased risk of developing these disorders.

The tremendous use of computer by the staff members, technicians, and students at King Abdulaziz University (KAU), by our experience, has been accompanied by increase in the number of attendances to University Medical Directorate (Services) with general, eye and vision, and musculoskeletal complaints. When this observation was brought to the attention of KAU officials, they urged and encouraged concerned personnel to study the nature of this problem and propose remedial actions.

Meanwhile, one of the first institutions that had applied computer technology in Saudi Arabia was the Saudi Airlines tickets' reservation offices (SAUDIA). It is considered to be one of most eligible areas to conduct a study regarding VDT health related problems. Putting this in mind, KAU urged concerned personnel to include it in the present study. Also, the Saudi Telecom Company (STC) works in Jeddah comprises nearly 430 VDT workstations where 360 operators and mostly 70 supervisors work for whole shifts. There have been some claims that these operators and supervisors suffer some general musculoskeletal and eye and vision complaints. Consequently, these works have been decided to be included in this study.

The objectives of the present study were

1. to evaluate the magnitude of the problem of inconveniences in the use of computers in KAU, SAUDIA, and STC, as well as the inconveniences in the computers' workstations,
2. to investigate computers' operators health complaints,
3. to investigate environmental and behavioral factors contributing to the occurrence of the complaints,
4. to propose remedial actions that might contribute to reducing these complaints.

## 2. Methodology

### 2.1. Study Population

Inventories of the computer workstations and operators in the different colleges and units of KAU, in the different departments and units of SAUDIA tickets' reservation offices, and in the different departments and units of STC head office in Jeddah had, primarily, been conducted to assess the magnitude of computer use there. The findings of the inventories are summarized in Table 1.

Representative random samples of 100 workstations, and operators (all males, since no females are employed there), were selected from each of the three institutions, considering that the selection of the sampled stations and operators had been affected by the readiness of the individual administrations and operators in the different departments and units to participate in the study. The selected stations are also presented in Table 1.

### 2.2. Studying Ergonomics of Workstations

A study form entitled “Ergonomics Rating of Computer Applications” was developed to assess the ergonomics status of the studied computer workstations. The form was designed after reviewing the ANS/HFES Committee document [6], and many computer's workstation evaluation checklists that had been tested and used by international institutions include

1. U.S. Department of Health and Human Services, Centers of Disease Control, and Prevention (CDC), Evaluation Checklist;
2. National Institute for Occupation Safety and Health (NIOSH) Ergonomics Work-Place Evaluations of Musculoskeletal Disorders Checklist;
3. U.S. Department of Labor, Occupational Safety, and Health Agency (OSHA) Computer Workstation Ergonomic Checklist;
4. University of California Computer Workstation Self-Evaluation Checklist;
5. California State University Ergonomics Evaluation Checklist;
6. Cornell University Ergonomics Checklist;
7. University of Virginia Library Ergonomics Evaluation Form;
8. Institute for Occupational Physiology at the University of Dortmund Checklist for Computer Workstation;
9. Atlantic Mutual Centennial Insurance Company Workstation Checklist.

The ergonomics score for the evaluation of the workstation is 43, distributed by the different components. Each component has certain number of scores, determining the maximum score of the component as shown in Table 2. Besides, 3 scores are allowed for the work organization and 4 scores for the training and provision of information, making a total score for the work at the specific workstation of 50, which is equivalent to 100% when scoring percentagewise.

Each score item is clearly presented to be answered by “Yes” or “No” to avoid any personal differences or any bias by the evaluators. The “Yes” answers are counted to represent the score out of 50, and some ten stations were evaluated to test the study from and found to be satisfactory for the conduct of the study. Furthermore, the evaluation of the workstations was carried out, only, by the authors for quality assurance of the data collection. The study form has been designed in four major sections including the following.

*Section (1).* It includes basic information of investigated organizations (colleges/units), particularly as related to presented services.

*Section (2).* It includes ergonomics rating of investigated workstations by checking the details of each component of the work place, including

1. desk, as related to space of desk top, layout of the desk, top equipment, desk top and distance from operator's eye, and existence of comfortable resting facility for operators' hands and rest;
2. seat, as related to dimensions, casters, operators' leg clearance, armrests, back rest, seat cushion, and seat comfort ability and stability;
3. footrest, as related to need, availability, and status of footrest;
4. display screen, as related to location, height and tilting of the monitor, distance from operator's eye, freedom of screen from glare and reflection, stability of image and freedom from flickering, ease to read characters, and possibility of adjusting screen brightness and contrast;
5. keyboard, as related to dimensions, location with reference to operator's hands and elbows, and exchanging operation between keyboard and mouse without operator's hand extension or twisting wrist;
6. mouse, as related to its location with reference to operator smooth running and operator's awareness of its details of operation and maintenance;
7. document holder, as related to need, availability, and status of the document holder;
8. space and room layout, as related to adequate access to work place, availability of space to maneuver the seat, work correct posture, availability of adequate space for equipment needed for work, location of monitor with reference to windows, freedom of work area from obstructions, and hazards of tripping and neatness of the work area;
9. task and posture, as related to freedom of operator's hands from phone while typing and resting his hand wrists;
10. illumination, as related to level of lighting, status of luminaries and illumination fixtures, use of blinds on windows, and background of the screen with surrounding environment;
11. noise and thermal environment, as related to level of quietness and status of air conditioning in work area.

*Section (3).* It includes work organization rating, by investigating work organization, work hours, rest pauses and noncomputer work assignment, and work load.

*Section (4).* It includes training and provision of information, by investigating operator's on-the-job and formal training, certainty of his use of software, keying habits, operator's capability of control of his workstation and work environment, and operator's adoption of good posture and avoiding visual fatigue at work.

### 2.3. Investigating Operators' Health Symptoms

A study form entitled “Impact of Computer Use on Operators” was developed to evaluate the effect of computer use on operator's health as reviewed and/or recommended by the NIOSH [1], WHO [5], and ANSI/HFES [6]. It is divided into four main sections as follows.

*Section (1).* It includes basic data, including name, gender, address, workstation, age, education, and smoking habit.

*Section (2).* It includes work data, including work type, duration of employment, formal training, work speed, daily hours of computer use, nature of computer use (continuous or intermittent), and work satisfaction.

*Section (3).* It includes health disorders before present work, including previous ailments or complaints of the musculoskeletal system and complaints of the eye and vision.

*Section (4).* It includes current symptoms, including the general complaints and their frequency, the eye and vision symptoms and their frequency, the maximum work hours before their occurrence and the time required for their release, and the musculoskeletal disorders and their location, description, frequency, and persistence, as well as the approached medical treatment and the sickness absenteeism as related to the work-related ailments.

### 2.4. Data Analysis

The collected data were visually inspected for fliers, then introduced into PC, and subjected to statistical analysis using Microsoft Excel 2007.

## 3. Results and Discussion

### 3.1. Ergonomics of the Workstations

**Figure 1:**
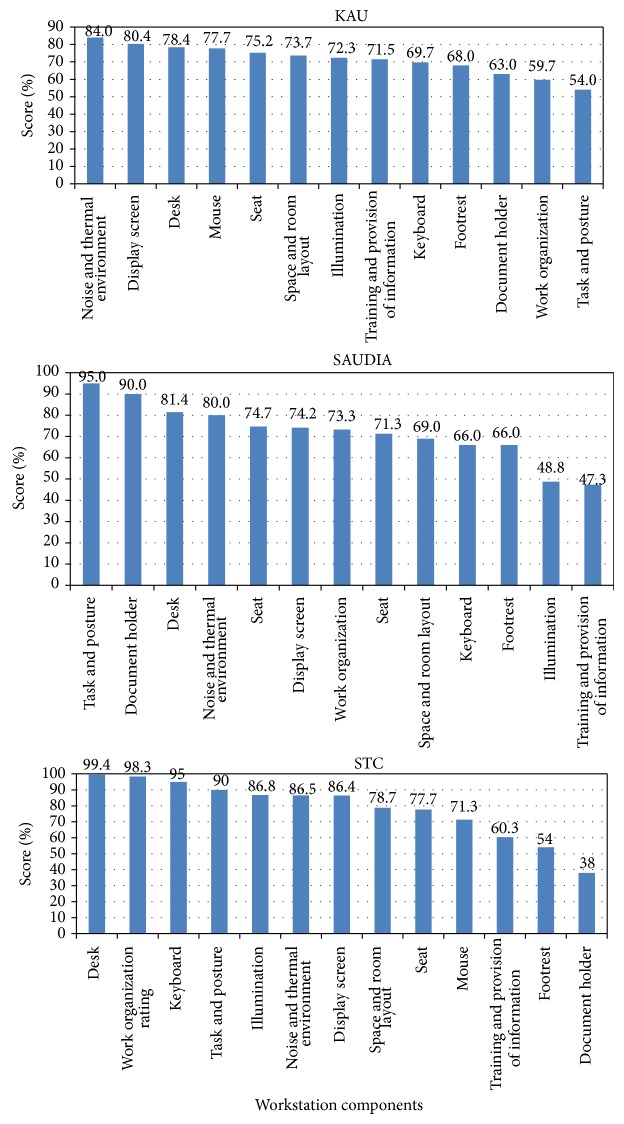
Average ergonomics scores of the examined workstation components.

**Figure 2:**
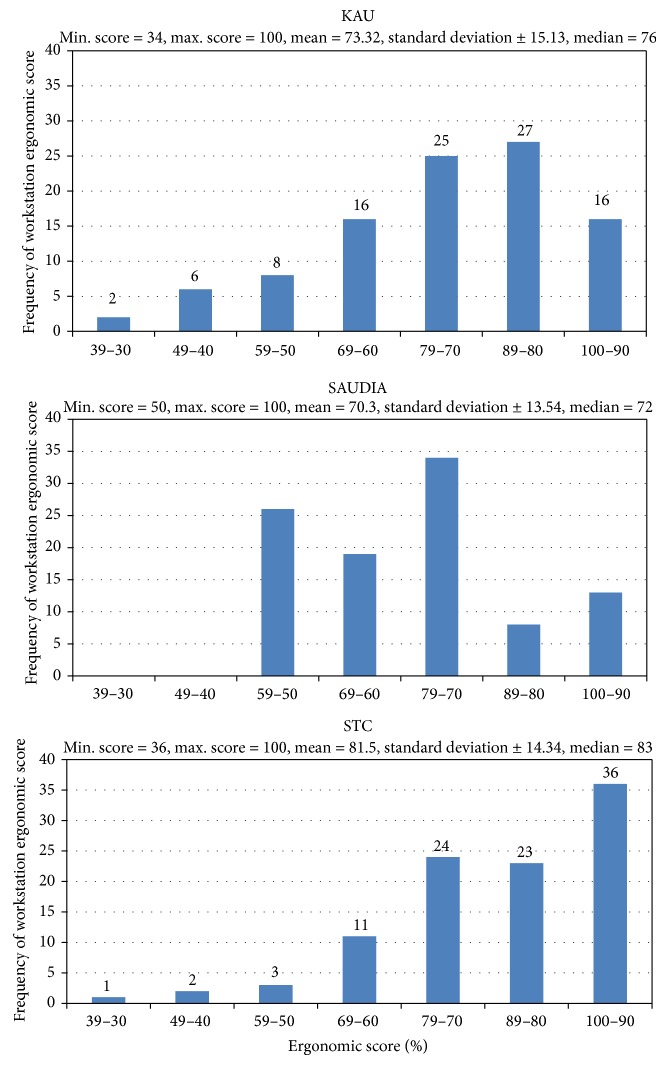
Distribution of the ergonomics scores of the examined workstation component.

The ergonomics scores of the studied workstations in the three institutions are illustrated in Table 3 and Figures 1 and 2. The average workstations score in STC has been rated very good (81.5 ± 14.34) which is considerably higher than the scores of both KAU and SAUDIA (73.3 + 15.13 and 70.3 ± 13.54, resp.) (Figure 2). This might be attributed to the relatively recent establishment of the workstations in STC in comparison to the other two study locations (KAU and SAUDIA). However, the score of the different components varies considerably in the three locations. For example, task and posture has been rated 95% and 90% at STC and SAUDIA, respectively, while it has been the lowest scored component at KAU (54%). Also, work organization has been rated the second highest (98.3%) at SAUDIA while it has been rated the second lowest at KAU (57.7%) and in the middle of the scores at SAUDIA (73.2%). These variations might be attributed to the differences of the type of work and pattern of computer use at the different study locations. The distribution of the ergonomics scores of the examined workstations might be considered to follow normal model but truncated (Figure 2).

### 3.2. Characteristics of the Work Population

The demographic and occupational characteristics of the studied populations of the computer users/operators in the three institutions are presented in Tables 4 and 5. The populations at the different study locations were mostly young, since 98% of the subjects in both KAU and STC, and 89% at SAUDIA, were younger than 50 years. However, the subjects of the study population at SAUDIA were relatively older since 27% of them were younger than 35 years in comparison to 80% at STC and 68% at KAU (Table 4). The average ages at the KAU, SAUDIA, and STC were 31.5, 39.7, and 30.3 years, respectively. Yet 78% and 73% of the populations at STC and KAU have been employed for less than 10 years, in comparison to 23% at SAUDIA that began using VDT earlier than the other two institutions (Table 5). The average durations of employment at KAU, SAUDIA, and STC were 7.1, 19,7, and 7.4 years, respectively. Meanwhile, the levels of education among KAU and STC populations were higher than the SAUDIA population. For example, 65% and 41% of KAU and STC populations received higher education in comparison to only 23% at SAUDIA population. Also, 16% of the KAU and 5% of the STC populations, respectively, received graduate education (Doctor and/or Master), while none of the subject at SAUDIA population had such education level.

Most of the study populations were nonsmokers (79%, 76%, and 62% of subjects at KAU, SAUDIA, and STC, resp.) and 26% of them at STC were light smoker (smoking index less than 200) that might be added to the proportion of the nonsmoker there to be 88%. This distribution might, however, be biased by the relatively young age of the examined subjects.

Considerable proportion of the populations either had no vision problems before employment (58%, 70%, and 58% at KAU, SAUDIA, and STC, resp.), or were short-sighted (30%, 23%, and 25%, resp.), while the rest were long-sighted or had other vision problems (14%, 7%, and 17%, resp.). Similarly, more than one half of the populations at the three study locations had no musculoskeletal symptoms before employment (59% at KAU, 62% at SAUDIA, and 55% at STC), while considerable proportions of the populations had neck pain (22% at KAU, 24% at SAUDIA, and 17% at STC). The rest of the populations had such symptom at one or more body locations.

More than one half of the population of KAU (52%) was either typist (23%) or involved in comprehensive office tasks (29%), while 40% of them were involved in data entry (22%) and data acquisition (22%). However, at SAUDIA, 77% of the populations were involved in data entry (54%) or data acquisition (23%) while 20% of them were involved in communication tasks and none of them was typist. Similarly, at STC, 86% of the populations were involved in communication tasks (53%) or data entry (33%), and none of them was typist. While 58% and 61% of the populations at KAU and SAUDIA, respectively, received on-the-job training only, and the rest received formal training for different periods, the opposite existed at STC, where 72% of the population received formal training for different periods, and only 28% of the population received on-the-job training only. Consequently, 61% of the populations at KAU and 70% at SAUDIA considered their work speed as average (56% and 70%, resp.) or slow (5% and 0%, resp.), while 45% of the population at STC considered their work speed as fast and 55% of them considered their work speed as either average (49%) or slow (6%).

Considerable proportions of the populations at KAU and STC used computer for 7, 8, or 9 hours per day (44% and 39%), while the whole population at SAUDIA (100%), and 53% of them at STC, used computer for 6 hours. On the other hand, 36% of the operators at KAU used computer for 3, 4, or 5 hrs. per day, while none of them at SAUDIA, and 9% of them at STC, operated computers for these shorter periods. However, only 53% of the SAUDIA population operated computer continuously in comparison to 85% of the STC and 61% of KAU populations. Meanwhile, mostly 70% of KAU (69%) and STC (68%) populations had rest pauses <25% of the work shift, and 22% of the two populations got rest pauses 30%–40% of the shift, while the whole SAUDIA population had 25%–29% of their shift as rest pauses, in comparison to 9% and 10% of the other two populations.

Eighty-two percent of the computer users in KAU, 72% of the operators at SAUDIA, and 60% of operators at STC were satisfied (and many were even very satisfied) at their work, particularly as related to their excellent satisfaction by their colleagues, work control, job attitude, and vigilance requirement, while the boredom from repetitive work and monotony and the work stress were the main causes of dissatisfaction among them, particularly the SAUDIA and STC populations (41%, 66%, and 65% at KAU, SAUDIA, and STC, resp.).

### 3.3. Operators' Health Complaints

The operators' health complaints are presented in Tables 6–9. Mostly one third of the operators (35%, 33%, and 27% of KAU, SAUDIA, and STC populations, resp.) was suffering from body fatigue, while 23%, 21%, and 37% of them were suffering from headache, such complaints occurred mostly sometimes among all the populations, however occurred to less extent, particularly among SAUDIA and STC operators. The lack of concentration occurred to less extent (for example, 8%, 6%, and 20% among KAU, SAUDIA, and STC populations, resp.), particularly and daily among SAUDIA and STC populations (Table 6).

Only 41% and 46% of KAU and STC populations, in comparison to 61% of SAUDIA population, reported eye and vision symptoms. The most predominant eye symptoms were eye redness, tearing, pain, and redness, and the most predominant vision symptoms were blurring, particularly for distance objects, as well as sensitivity to light (Table 7).

Thirty percent, 49%, and 39% of the KAU, SAUDIA, and STC populations were free from musculoskeletal symptoms. The main occurring symptoms were aching, tingling, numbness, pain, and stiffness, which occurred, mostly sometimes, and, to a less extent, often (Table 8). The highest incidences of the symptoms were at the operators' higher and lower back, neck and shoulder, arm, elbow, forearm, and fingers and then at the lower limbs (buttock to foot) (Table 9).

### 3.4. Factors Affecting Incidence of Complaints

The effects of age and duration of employment (i.e., work) on the incidence of operators' health complaints are shown in Tables 10 and 11. There has been general trend of increasing the different complaints by age, particularly among those exceeding 35 years of age (Table 10). This observation is further confirmed in Table 11, where the operators working for >10 years had, generally, the highest incidences of the general and the eye and vision complaints, as well as the incidences of other complaints, but to a less extent.

The impact of the ergonomics score of the workstation on the incidence of operators' complaints is shown in Table 12, where there has been a trend of decrease in the incidence of operators' general complaints, eye and vision complaints, and musculoskeletal complaints, particularly the extremities and the lower trunk complaints, by the increase of the ergonomics score of their workstations.

Out of the many factors considered for their effects on the incidences of the operators' complaints and symptoms, the smoking habit, the type of work, workers satisfaction, and the operators' history of musculoskeletal complaints and of eye and vision before joining present work showed some effects as indicated in Tables 13–17. Smoking appears to have some effect on increasing the incidences of the general and eye and vision complaints, particularly among KAU computer users and SAUDIA operators, and on the lower extremities and lower trunk complaints, to some extent (Table 13).

It is worth noting that the lowest eye and vision complaints occurred among the operators who had the lowest level of education (i.e., middle education), which might be interpreted by their relatively lower involvement in vision tasks than the operators having higher levels of education.

As related to the impact of type of work on the incidence of complaints, results in Table 14 show that the operators who were involved in communication tasks in KAU, and in data acquisition in SAUDIA, had the lowest general, eye and vision, neck and shoulder, lower extremities, and lower trunk complaints, as well as those involved in comprehensive activities among all the populations, meanwhile showing the highest freedom from all complaints. It may be noted that the numbers of operators involved in these activities (KAU communication tasks and SAUDIA and STC comprehensive tasks = 8, 3, and 5, resp.) were the lowest among all worker involved in other types of activities which might have some effect on the results.

Nevertheless, the work satisfaction showed clear impact on the incidence of health complaints among the examined computer users, where the percentages of those who were free from complaints got higher by the improvement of work satisfaction (Table 15); meanwhile, the lowest incidences of mostly all the complaints were the lowest among the very satisfied operators, particularly the SAUDIA and STC operators.

The history of previous ailments among computer users/operators, also, had some impact on the reported complaints among them, where the percentages of the present complaints among the subjects who had no previous ailments were less than among the other subjects reporting related ailments' history (Tables 16–18).

## 4. Conclusions 

The average ergonomics score at STC was 81% which may be considered as a good level. However, and unexpectedly, the average ergonomics scores at KAU and SAUDIA were only 73.3% and 70.3%, respectively. It had been anticipated that the average ergonomics scores for the computer workstations existing in leading institutions like KAU and SAUDIA should be considerably higher.

Although the examined populations in KAU and STC were relatively young and, consequently, had relatively short employment work duration, were relatively highly educated, had relatively low smoking index and low history of ailments before employment, had some type of on-the-job and/or formal training, mostly use computer daily for <7 hours and continuously getting rest pauses, and were mostly satisfied at work, yet they had somewhat high incidences of general complaints (e.g., body fatigue, headache, and lack of concentration), vision complaints, and musculoskeletal complaints. However, within SAUDIA population, surprisingly, the highest health complaints were among the youngest operators, who also had the lowest duration of computer work, as well as among those who had on-the-job and/or formal training; meanwhile, no systematic effect of the workstations' ergonomic scores on the incidence of the complaints was observed. These anomalies might be attributed to having some of the operators who developed complaints there left or changed their work.

Naturally, the operators who were satisfied by their work and those who were conducting comprehensive works (i.e., variable types of work) as well as those who had no, or inconsiderable, history of previous ailments had the least incidence of the health complaints.

Meanwhile, higher incidences of the complaints existed among the smoking operators and those who did not work continuously with computer, as well as those who rated themselves as fast operating.

In summary, the incidence of the various complaints had been demonstrated, generally, to increase by (a) the decrease in the ergonomics score of the workstations, (b) the progress of age and duration of employment, (c) the increase of smoking habit, (d) the continuous daily use of computer, (e) the lack of work satisfaction, and (f) the history of operators' previous ailments. However, unexpectedly, no effect could be demonstrated of the operators' formal training and the daily hours of computer use, on the incidences of the complaints.

It is anticipated that the incidences of the different complaints among the examined population increased by their progress in the duration of work. Therefore, it is recommended that rapid actions should be taken to improve the ergonomics of the computer workstations. The improvement of each workstation should be considered separately with reference to the evaluation checklist of its individual components.

Setting up training programs for computer operators to efficiently use their computers and optimize their posture and movements inside their computer workstations based on ergonomics principles is highly recommended. Also, motivation of workers to learn about computer work-related health disorders, their causes, etiology, preferable postures and movements, and the role of fitness exercise, and encouraging them to take rest pauses within their work shifts, all are recommended.

It is recommended to conduct preplacement examination for computers' operators to exclude subjects with history of ailments that might be aggravated by computer use and to have available health baseline for the employed subjects as well as periodical medical examination (annually or each two years) to assure normal health background and to early discover any deviation from normality.

Finally, the study recommends extending the research to cover the sectors of computer and VDTs users, particularly those employed by small offices and medium-size enterprises where it is anticipated to have ergonomics poorly designed workstations. Also, particular interest may be forwarded to investigating the presently studied complaints among the female computer users in KSA.

## Figures and Tables

**Table 1:** Existing computer workstations and operators in the different units of KAU, SAUDIA, and STC and the sample selected for the study.

Institution	Units	Existing service			Sample		
		Workstation	Supervisor	Operator	Workstation	Supervisor	Operator
King Abdulaziz University (KAU)	(i) Higher administration, including Deanship of Admission and Registration and Deanship of Student Affairs	301			14		
	(ii) Deanship of Information Technology	114			16		
	(iii) Deanship of Library Affairs	73					
	(iv) Faculty of Economics and Administration	96			9		
	(v) Faculty of Sciences	86			9		
	(vi) Faculty of Engineering	130			16		
	(vii) Faculty of Medicine and University Hospital	34			17		
	(viii) Faculty of Arts and Humanities	81			14		
	(ix) Faculty of Earth Sciences	63			5		
	(x) Faculty of Environmental Designs	41					
	(xi) Faculty of Marine Sciences	8					
	(xii) Faculty of Meteorology, Environment and Arid Land Agriculture	16					
	**Total**	**1043**			**100**		

Saudi Airlines Ticket Reservation (SAUDIA)	(i) Central Control for Africa and Europe Flights	20			15		
	(ii) Central Control for Local and Gulf Flights	20			15		
	(iii) Central Control for Asia and Middle East Flights	10			5		
	(iv) Record and Follow-up Department	20			10		
	(v) Customer Services Department	165			55		
	**Total**	235			**100**		

Saudi Telecom Company (STC)	(i) English Call Services Department		15	90		4	16
	(ii) Help Services Department		24	120		8	27
	(iii) Other Services Department		30	150		10	35
	**Total**		69	360		22	**78**

**Table 2:** Distribution of the ergonomics scores of the different components of the studied workstations.

Workstation component	Maximum score
(1) Desk	5
(2) Seat	6
(3) Footrest	1
(4) Display screen	8
(5) Keyboard	3
(6) Mouse	3
(7) Document holder	2
(8) Space and room layout	7
(9) Task and posture	2
(10) Illumination	4
(11) Noise and thermal environment	2

Total scores	43

**Table 3:** Positive ergonomics components of the examined workstations.

Number	Ergonomics components	KAU∗ (*N* = 100)		SAUDIA∗∗ (*N* = 100)		STC∗∗∗ (*N* = 100)	
		Number of positives	Average	Number of positives	Average	Number of positives	Average
I	Noise and thermal environment						
1	Quietness	75	84.0	78	81.5	83	86.5
2	Air-conditioning	93		85		90	

II	Display screen						
3	Monitor location	71	80.4	70	75.4	97	87.4
4	Monitor top	80		100		99	
5	Monitor distance from eye	71		100		98	
6	Monitor tilting	75		72		97	
7	Glare and reflection	68		60		70	
8	Image stability	91		67		80	
9	Ease of reading	95		68		74	
10	Brightness and contrast	92		66		84	

III	Desk						
11	Space	81	78.4	100	81.4	100	99.4
12	Layout	85		85		99	
13	Distance from eye	74		86		100	
14	Room for leg	93		65		100	
15	Hand/wrist	59		71		98	

IV	Mouse						
16	Distance from hand	83	77.7	75	71.3	72	71.3
17	Run	76		78		76	
18	Operator's familiarity	74		61		66	

V	Seat						
19	Height	89	75.3	100	74.7	99	77.7
20	Dimensions	78		72		95	
21	Armrest	76		75		77	
22	Backrest	64		79		59	
23	Pad (foam)	71		60		63	
24	Comfort and stability	73		62		73	

VI	Space and room layout						
25	Adequate access	90	73.7	65	68.9	24	78.7
26	Space around seat	86		100		100	
27	Layout	80		61		93	
28	Location of equipment	62		61		88	
29	Monitors' positions	51		66		100	
30	Obstructions and hazards	75		60		100	
31	Housekeeping	72		69		46	

VII	Illumination						
32	Lighting level	91	72.3	55	48.8	60	86.8
33	Luminaries	66		46		99	
34	Effectiveness	61		43		97	
35	Background behind screens	71		51		91	

VIII	Training and provision of information						
36	Use of software	75	71.5	46	47.3	76	60.3
37	Habit keying	73		59		66	
38	Adjustment	74		43		65	
39	Good posture and visual fatigue	64		41		34	

IX	Keyboard						
40	Distance	69	69.7	66	66.0	98	95.0
41	Width	73		69		75	
42	Height and key angle	67		63		92	

X	Footrest						
43	Compression of thigh	68	68.0	65	65.0	54	54.0

XI	Document holder						
44	Need	64	63.0	90	90.0	39	38.0
45	Balance of head posture	62		90		37	

XII	Work organization rating						
46	Breaks	79	59.7	100	73.3	88	89.3
47	Urgent peaks and interruptions	40		55		83	
48	Over time	60		65		97	

XIII	Task and posture						
49	Phoning while typing	33	54.0	90	95.0	99	90.0
50	Typing posture	75		100		81	
Total average score		** **	**72.3**	** **	**71.1**	** **	**81.0**

∗KAU = King Abdulaziz University.

∗∗SAUDIA = Saudi Airlines.

∗∗∗STC = Saudi Telecom Company.

**Table 4:** Demographic characteristics of the study population.

Demographic characteristics	Frequency		
	KAU (*N* = 100)	SAUDIA (*N* = 100)	STC (*N* = 100)
Age (years)			
20–24	24	9	19
25–29	30	8	47
30–34	14	10	14
35–39	15	18	7
40–44	10	20	8
45–49	5	24	3
50–54	2	9	2
>55	0	2	0
Education			
Middle	6	2	2
Secondary (general)	21	71	55
Secondary (technical)	8	4	2
High (technical)	19	2	13
High (administrative)	30	21	23
Graduate (master + doctor)	16	0	5
Smoking index			
** **Nonsmokers	**79**	**76**	**62**
<100	6	5	17
100–199	3	2	9
200–399	2	4	5
400–500	5	3	3
>600	5	10	4
Vision symptoms prior to present work∗			
** **None	**58**	**70**	**58**
Short-sighted	30	23	25
Long-sighted	7	2	10
Others	7	5	7
Musculoskeletal symptoms prior to present Work∗			
** **None	**59**	**62**	**55**
Neck pain	22	24	17
Shoulder and/or arms pain	11	11	4
Lower trunk pain	13	23	16
Thigh and leg pain	5	8	4
Others	1	1	4

∗The same subject might have more than one symptom occurring at different frequencies.

**Table 5:** Occupational characteristics of the study population.

Occupational characteristics	Frequency		
	KAU (*N* = 100)	SAUDIA (*N* = 100)	STC (*N* = 100)
Duration of employment (years)			
<1	12	7	7
1-2	23	5	24
3-4	20	4	17
5–9	18	7	30
10–14	11	7	5
15–19	7	14	9
20–24	5	20	5
25–29	3	16	3
30–34	1	16	0
≥35	0	4	0
Type of work			
Data entry	22	54	33
Data acquisition	18	23	9
Typist	23	0	0
Communication task	8	20	53
Comprehensive office tasks	29	3	5
Duration of formal training (days) On-the-job training only	**58**	**61**	**28**
<50	12	19	24
50–99	5	8	14
100–199	11	2	20
200–299	4	2	4
300–399	4	4	1
400–499	3	0	1
≥500	3	4	8
Work speed			
Fast	39	30	45
Average	56	70	49
Slow	5	0	6
Computer use (hrs/day)			
3	15	0	2
4	12	0	3
5	9	0	3
6	20	100	53
7	14	0	1
8	22	0	17
9	8	0	21
Nature of daily work on computer			
Continuous	61	53	85
Intermittent	39	47	15
Rest pauses of work shift (%)			
5–9	10	0	12
10–14	22	0	19
15–19	18	0	16
20–24	19	0	21
25–29	9	100	10
30–34	9	0	11
35–39	7	0	6
≥40	6	0	5
Elements of work satisfaction			
Satisfaction by foreman and colleagues interrelations	100	99	99
Satisfaction by absence work stress	68	60	61
Satisfaction of work control	96	94	96
Satisfaction of job attitude	92	81	82
Satisfaction by vigilance requirement	94	100	94
Satisfaction by nature of work	73	85	55
Satisfaction by absence of repetitive work and monotony	59	34	35
Evaluation of work satisfaction∗			
Very satisfied	39	35	29
Satisfied	43	37	31
Satisfied to some extent	10	14	27
Not satisfied	**8**	**14**	**13**

∗Percent of duration(s) of rest pauses to duration of work shift.

**Table 6:** Incidence of work-related general symptoms among the examined computer users/operators.

Symptoms	Frequency														
	KAU (*N* = 100)					SAUDIA (*N* = 100)					STC (*N* = 100)				
	None	Some-times	Often	Daily	Total affected∗	None	Some-times	Often	Daily	Total affected∗	None	Some-times	Often	Daily	Total affected∗
Headache	77	17	6	0	23	79	11	6	4	21	63	25	11	1	37
General body fatigue	65	31	4	0	35	67	17	12	4	33	73	17	9	1	27
Lack of concentration	92	7	1	0	8	94	1	3	2	6	80	10	8	2	20
Total	**46**	45	9	0	**54**	**60**	22	12	6	**40**	**97**	1	1	1	**3**

∗The same subject may have more than one symptom occurring at different frequencies.

**Table 7:** Incidence of work-related eye and vision symptoms among the examined computer users/operators.

Symptoms	Frequency														
	KAU (*N* = 100)					SAUDIA (*N* = 100)					STC (*N* = 100)				
	None	Some-times	Often	Daily	Total affected∗	None	Some-times	Often	Daily	Total affected∗	None	Some-times	Often	Daily	Total affected∗
Eye															
Eye discomfort	85	12	3	0	15	88	8	4	0	12	91	7	1	1	9
Aches	91	6	3	0	9	96	3	1	0	4	97	1	2	0	3
Pain	95	4	1	0	5	95	2	3	0	5	92	5	3	0	8
Redness	91	6	2	1	9	90	8	1	1	10	93	5	2	0	7
Irritation and itching	93	4	3	0	7	94	3	3	0	6	96	2	2	0	4
Burning	88	9	3	0	12	93	5	2	0	7	95	3	2	0	5
Tearing	82	14	4	0	18	92	3	4	1	8	91	7	1	1	9
Dryness	96	3	1	0	4	92	5	2	1	8	96	3	1	0	4
Vision															
Blurred: close objects	92	6	2	0	8	93	3	3	1	7	95	3	2	0	5
Blurred: distant objects	88	9	3	0	12	86	8	5	1	14	91	6	3	0	9
Sensitivity to light	86	10	3	1	14	92	3	4	1	8	84	14	2	0	16
Double flickering	96	4	0	0	4	93	2	4	1	7	97	2	1	0	3
Double vision	99	1	0	0	1	94	3	2	1	6	93	4	3	0	7
Change in color perception	98	2	0	0	2	99	1	0	0	1	97	2	1	0	3
Others	98	1	1	0	2	99	0	1	0	1	0	0	0	0	0
All eye and vision symptoms	**41**	48	9	2	**59**	**61**	22	12	5	**39**	**46**	14	15	25	**54**

∗The same subject may have more than one symptom occurring at different frequencies.

**Table 8:** Incidence of work-related musculoskeletal symptoms among the examined computer users/operators.

Symptoms	Frequency														
	KAU (*N* = 100)					SAUDIA (*N* = 100)					STC (*N* = 100)				
	None	Some-times	Often	Daily	Total affected∗	None	Some-times	Often	Daily	Total affected∗	None	Some-times	Often	Daily	Total affected∗
Aching	73	18	8	1	27	84	9	6	1	16	69	20	10	1	31
Tingling	84	11	5	0	16	93	5	2	0	7	89	9	3	0	12
Numbness	93	5	1	1	7	92	2	5	1	8	88	4	7	0	11
Burning	95	3	2	0	5	98	0	1	1	2	96	1	3	0	4
Paleness	99	0	0	1	1	100	0	0	0	0	99	1	0	0	1
Swelling	98	1	0	1	2	97	1	1	1	3	97	0	3	0	3
Pain	92	2	3	3	8	84	8	6	2	16	84	6	8	2	16
Stiffness	93	4	2	1	7	91	3	4	2	9	89	4	7	0	11
Cramping	98	1	1	0	2	96	1	2	1	4	98	1	1	0	2
Total	**30**	47	17	6	**70**	**49**	27	21	3	**51**	**39**	47	10	4	**61**

∗The same subject may have more than one symptom occurring at different frequencies.

**Table 9:** Locations and persistency of the work-related musculoskeletal symptoms among the examined computer users/operators.

Symptoms	Frequency																	
	KAU (*N* = 100)						SAUDIA (*N* = 100)						STC (*N* = 100)					
	None	One hr	One day	One week	One month to 1 year	Total∗	None	One hr	One day	One week	One month to 1 year	Total∗	None	One Hr	One day	One week	One month to 1 year	Total∗
Neck	78	6	10	3	3	22	77	7	10	4	2	23	81	10	5	2	2	19
Shoulder	27	7	13	5	0	28	75	10	10	1	4	25	83	8	6	3	0	17
Arm and elbow	89	5	5	0	1	11	92	1	5	0	2	8	97	1	1	1	0	3
Forearm	91	1	6	1	1	9	90	4	3	0	3	10	98	3	1	0	0	2
Fingers	88	4	7	0	1	12	91	2	5	0	2	9	91	3	4	2	0	9
Higher back	75	7	16	1	1	25	82	5	10	2	1	18	84	3	8	5	0	16
Lower back	67	8	19	2	4	33	82	9	6	1	2	18	70	10	14	5	1	30
Buttock	97	0	2	0	1	3	87	5	7	1	0	13	97	2	1	0	0	3
Thigh	96	1	2	1	0	4	87	4	7	0	2	13	93	3	3	0	1	7
Knee	93	3	3	0	1	7	92	4	3	1	0	8	89	4	5	2	0	11
Leg	93	2	3	1	1	7	94	3	1	1	1	6	95	1	2	2	0	5
Foot	94	3	1	1	1	6	91	4	4	0	1	9	94	1	2	2	1	6
All symptoms	**30**	27	30	9	4	**70**	**49**	25	18	4	4	**51**	**39**	23	24	10	4	**61**

∗The symptoms may occur in more than one location at the same frequencies.

**Table 10:** Incidence of complaints as related to age of computer users/operators.

Age (year)	Number of operators	Ergonomic score mean (SD)	Duration of employment (year) mean (SD)	Computer use (hours/day) mean (SD)	Complaints *N* (%)						
					None	General	Eye and vision	Neck and shoulder	Upper extremity	Lower extremity	Trunk
King Abdulaziz University computer users											
20–29	54	37.4	2.6	6.4	7	24	31	26	16	8	25
		(5.0)	(1.1)	(1.5)	(13.0)	(44.4)	(57.4)	(48.1)	(29.6)	(14.8)	(46.3)
30–39	29	36.7	8.8	5.8	5	17	15	16	12	4	16
		(6.3)	(3.5)	(2.0)	(17.2)	(58.6)	(51.7)	(55.2)	(41.4)	(13.8)	(55.2)
40+	17	34.9	17.6	6.0	3	10	11	9	4	3	7
		(6.5)	(7.2)	(2.5)	(17.6)	(58.8)	(64.7)	(52.9)	(23.5)	(17.6)	(41.2)

Saudi Airlines Ticket reservation operators											
20–29	17	69.1	2.0	6.0	4	7	8	7	4	3	8
		(7.7)	(1.3)	(0.0)	(23.5)	(41.2)	(47.1)	(41.2)	(23.5)	(17.6)	(47.1)
30–39	28	71.6	14.9	6.0	11	13	10	10	7	6	7
		(11.0)	(3.1)	(0.0)	(39.3)	(46.4)	(35.7)	(35.7)	(25.0)	(21.4)	(25.0)
40+	55	72.8	26.4	6.0	26	20	21	18	11	14	20
		(13.6)	(4.9)	(0.0)	(47.3)	(36.4)	(38.2)	(32.7)	(20.0)	(25.5)	(36.4)

Saudi Telecom Co. computer operators											
20–29	66	78.4	3.2	7.2	12	40	38	34	19	32	7
		(9.8)	(1.4)	(1.6)	(18.2)	(60.6)	(57.6)	(51.5)	(28.8)	(48.5)	(10.6)
30–39	21	82.2	8.7	7.4	3	14	13	12	5	10	6
		(10.9)	(3.1)	(1.5)	(14.3)	(66.7)	(61.9)	(57.1)	(12.4)	(47.6)	(28.6)
40+	13	95.9	21.6	6.6	1	9	8	5	2	8	0
		(4.1)	(3.2)	(1.6)	(7.7)	(69.2)	(61.5)	(38.5)	(15.4)	(61.5)	(0.0)

**Table 11:** Incidence of complaints as related to duration of work.

Duration of employment (year)	Number of operators	Ergonomic score mean (SD)	Age (year) mean (SD)	Computer use (hours/day) mean (SD)	Complaints *N* (%)						
					None	General	Eye and vision	Neck and shoulder	Upper extremity	Lower extremity	Trunk
King Abdulaziz University computer users											
≤2	35	37.6	25.8	6.5	6	14	17	16	7	4	14
		(4.4)	(2.7)	(1.7)	(17.1)	(40.0)	(48.6)	(45.7)	(20.0)	(11.4)	(40.0)
3–9	38	36.2	29.3	6.4	5	21	22	22	14	6	22
		(6.3)	(3.5)	(1.9)	(13.2)	(55.3)	(57.9)	(57.9)	(36.8)	(15.8)	(57.9)
≤10	27	36.4	42.3	5.8	4	16	18	13	10	5	12
		(7.9)	(4.5)	(2.2)	(14.8)	(59.3)	(66.7)	(48.1)	(37.0)	(18.5)	(44.4)

Saudi Airlines Ticket reservation operators											
≤2	12	69.7	23.3	6.0	3	5	6	5	2	3	5
		(8.2)	(1.6)	(0.0)	(25.0)	(41.7)	(50.0)	(41.7)	(16.7)	(25.0)	(41.7)
3–9	11	68.9	30.6	6.0	4	5	3	4	2	1	4
		(7.4)	(2.1)	(0.0)	(36.4)	(45.5)	(27.3)	(36.4)	(18.2)	(9.1)	(36.4)
≤10	77	71.9	43.1	6.0	34	30	30	26	18	19	25
		(13.7)	(2.9)	(0.0)	(44.2)	(39.0)	(39.0)	(33.8)	(23.4)	(24.7)	(32.5)

Saudi Telecom Co. computer operators											
≤2	31	76.7	25.2	7.2	6	17	18	17	9	14	6
		(13.5)	(2.2)	(1.9)	(19.4)	(54.8)	(58.1)	(54.8)	(29.0)	(45.2)	(19.4)
3–9	47	80.3	27.8	7.1	8	28	24	21	13	26	4
		(7.7)	(1.9)	(1.5)	(17.0)	(59.6)	(51.1)	(44.7)	(27.7)	(55.3)	(8.5)
≤10	22	90.3	40.8	7.1	2	18	17	13	4	10	3
		(13.2)	(5.4)	(1.8)	(9.1)	(81.8)	(77.3)	(59.1)	(18.2)	(45.5)	(13.6)

**Table 12:** Incidence of complaints as related to ergonomic score of workstation.

Ergonomic score	Number of operators	Age (year) mean (SD)	Duration of employment (year) mean (SD)	Computer use (hours/day) mean (SD)	Complaints *N* (%)						
					None	General	Eye and vision	Neck and shoulder	Upper extremity	Lower extremity	Trunk
King Abdulaziz University computer users											
<60	18	32.7	7.7	5.5	2	11	12	10	9	5	8
		(6.9)	(4.3)	(1.6)	(11.1)	(61.1)	(66.7)	(55.6)	(50.0)	(27.8)	(44.4)
60–79	41	31.7	7.2	6.5	7	20	22	19	11	7	21
		(7.6)	(6.1)	(1.5)	(17.1)	(48.8)	(48.8)	(46.3)	(26.8)	(17.1)	(51.2)
80+	27	30.1	6.5	6.1	6	20	26	22	11	4	19
		(5.8)	(4.4)	(2.1)	(14.6)	(48.8)	(63.4)	(53.7)	(26.8)	(9.8)	(46.3)

Saudi Airlines Ticket reservation operators											
<60	21	40.4	20.3	6.0	8	10	10	6	4	4	8
		(10.2)	(11.4)	(0.0)	(38.1)	(47.6)	(47.6)	(28.6)	(19.0)	(19.0)	(38.1)
60–79	57	38.9	18.4	6.0	23	20	20	21	10	14	21
		(6.7)	(7.9)	(0.0)	(40.4)	(35.1)	(35.1)	(36.8)	(17.5)	(24.6)	(36.8)
80+	22	40.0	5.5	6.0	10	10	9	8	8	5	5
		(4.5)	(5.2)	(0.0)	(45.5)	(45.5)	(40.9)	(36.4)	(36.4)	(22.7)	(22.7)

Saudi Telecom Co. computer operators											
<60	6	26.1	3.6	7.8	1	3	3	3	2	4	3
		(2.1)	(2.8)	(0.9)	(16.7)	(50.0)	(50.0)	(50.0)	(33.3)	(66.7)	(50.0)
60–79	35	27.0	3.8	6.8	5	24	19	19	10	19	8
		(2.6)	(2.0)	(1.3)	(14.3)	(68.6)	(54.3)	(54.3)	(28.6)	(54.3)	(22.9)
80+	59	31.9	8.8	7.2	8	36	37	29	14	27	2
		(5.8)	(5.8)	(1.6)	(13.6)	(61.0)	(62.7)	(49.2)	(23.7)	(45.8)	(3.4)

**Table 13:** Incidence of complaints as related to smoking habits.

Smoking habit	Number of operators	Ergonomic score mean (SD)	Age (year) mean (SD)	Duration of employment (year) mean (SD)	Computer use (hours/day) mean (SD)	Complaints *N* (%)						
						None	General	Eye and vision	Neck and shoulder	Upper extremity	Lower extremity	Trunk
King Abdulaziz University computer users												
Nonsmokers	79	37.2	30.7	6.3	6.2	12	37	22	40	24	10	37
		(7.6)	(9.1)	(6.6)	(2.2)	(15.2)	(46.8)	(27.8)	(50.6)	(30.4)	(12.7)	(46.8)
Smokers	21	35.3	34.2	9.0	6.2	3	14	17	11	7	5	11
		(7.4)	(9.3)	(7.9)	(3.1)	(14.3)	(66.7)	(81.0)	(52.4)	(33.3)	(23.8)	(52.4)

Saudi Airlines Ticket Reservation operators												
Nonsmokers	75	72.2	39.5	18.9	6.0	32	27	26	21	15	15	21
		(13.8)	(9.1)	(10.7)	(0.0)	(42.7)	(36.0)	(34.7)	(28.0)	(20.0)	(20.0)	(28.0)
Smokers	25	69.0	40.4	20.9	6.0	9	13	13	14	7	8	13
		(10.4)	(9.3)	(9.9)	(0.0)	(36.0)	(52.0)	(52.0)	(56.0)	(28.0)	(32.0)	(52.0)

Saudi Telecom Co. computer operators												
Nonsmokers	62	81.8	29.0	6.7	7.0	9	38	35	34	16	33	8
		(15.5)	(6.5)	(6.6)	(2.1)	(14.5)	(61.3)	(56.5)	(54.8)	(25.8)	(53.2)	(12.9)
Smokers	38	81.0	30.3	7.5	8.7	7	25	24	17	10	17	5
		(12.5)	(8.4)	(8.3)	(8.6)	(18.4)	(65.8)	(63.2)	(44.7)	(26.3)	(44.7)	(13.2)

**Table 14:** Incidence of complaints as related to type of work.

Type of work	Number of operators	Ergonomic score mean ± SD	Age (year) mean ± SD	Duration of employment (year) mean ± SD	Computer use (hours/day) mean ± SD	Complaints *N* (%)						
						None	General	Eye and vision	Neck and shoulder	Upper extremity	Lower extremity	Trunk
King Abdulaziz University computer users												
Data entry	** 22**	37.5	29.4	5.6	7.0	3	14	10	12	7	1	10
		(6.6)	(9.4)	(7.8)	(2.1)	(13.6)	(63.4)	(45.5)	(54.5)	(31.8)	(4.5)	(45.5)
Typist	** 23**	36.9	33.8	9.8	7.1	3	17	14	14	8	3	10
		(8.2)	(9.3)	(6.7)	(3.0)	(13.0)	(73.9)	(60.9)	(60.9)	(34.8)	(13.0)	(43.5)
Data acquisition	** 18**	35.4	33.5	8.6	6.0	1	13	12	8	3	4	11
		(6.3)	(10.6)	(7.3)	(2.0)	(5.6)	(72.2)	(66.7)	(44.4)	(16.7)	(22.2)	(61.1)
Communication task	** 8**	37.0	28.4	4.1	6.6	3	3	3	3	2	0	3
		(5.8)	(5.9)	(5.9)	(5.5)	(37.5)	(37.5)	(37.5)	(37.5)	(25.0)	(0.0)	(37.5)
Comprehensive	** 29**	36.8	30.0	5.4	4.8	4	10	20	13	4	6	14
		(9.2)	(7.5)	(6.0)	(1.9)	(13.8)	(34.5)	(69.0)	(44.8)	(13.8)	(20.7)	(48.3)

Saudi Airlines Ticket reservation operators												
Data entry	** 54**	69.6	38.3	17.2	6.0	11	28	26	24	14	15	23
		(12.0)	(10.4)	(11.1)	(0.0)	(20.4)	(51.8)	(48.1)	(44.4)	(25.9)	(27.8)	(42.6)
Data acquisition	** 23**	77.8	43.3	26.2	6.0	17	3	4	3	2	2	2
		(15.8)	(6.1)	(7.1)	(0.0)	(73.9)	(13.0)	(17.4)	(13.0)	(8.7)	(8.7)	(8.7)
Communication task	** 20**	66.6	36.7	16.1	6.0	10	9	9	8	6	6	9
		(9.6)	(8.6)	(10.8)	(0.0)	(50.0)	(45.0)	(45.0)	(40.0)	(30.0)	(30.0)	(45.0)
Comprehensive	** 3**	82.0	39.0	19.3	6.0	3	0	0	0	0	0	0
		(14.0)	(1.7)	(4.1)	(0.0)	(100)	(0.0)	(0.0)	(0.0)	(0.0)	(0.0)	(0.0)

Saudi Telecom Co. computer operators												
Data entry	** 33**	83.4	31.7	8.9	7.2	7	22	22	18	6	16	2
		(12.7)	(7.9)	(8.1)	(1.8)	(21.2)	(66.7)	(66.7)	(54.5)	(18.2)	(48.5)	(6.1)
Data acquisition	** 9**	85.1	30.0	5.3	9.3	0	6	7	7	3	3	1
		(8.3)	(3.7)	(4.7)	(2.0)	(0.0)	(66.7)	(77.8)	(77.8)	(33.3)	(33.3)	(11.1)
Communication task	** 53**	79.4	28.2	5.4	6.9	8	32	28	26	14	30	9
		(15.7)	(7.0)	(6.8)	(2.1)	(15.1)	(60.4)	(52.8)	(49.1)	(26.4)	(56.6)	(17.0)
Comprehensive	** 5**	85.2	34.8	12.8	4.4	1	3	2	0	3	1	1
		(17.1)	(7.3)	(9.3)	(2.2)	(20.0)	(60.0)	(40.0)	(0.0)	(60.0)	(20.0)	(20.0)

**Table 15:** Incidence of complaints as related to work satisfaction.

Work satisfaction	Number of operators	Ergonomic score mean ± SD	Age (year) mean ± SD	Duration of employment (year) mean ± SD	Computer use (hours/day) mean ± SD	Complaints *N* (%)						
						None	General	Eye and vision	Neck and shoulder	Upper extremity	Lower extremity	Trunk
King Abdulaziz University computer users												
Very satisfied	39	38.0	33.7	8.5	6.4	8	18	21	21	7	3	14
		(6.5)	(10.2)	(8.2)	(2.7)	(20.5)	(46.2)	(53.8)	(53.8)	(17.9)	(7.7)	(35.9)
Satisfied	43	36.6	30.1	5.3	6.0	6	21	22	18	10	7	24
		(8.2)	(9.2)	(5.3)	(2.2)	(14.0)	(48.8)	(51.2)	(41.9)	(23.3)	(16.3)	(55.8)
Satisfaction to some extent	10	33.5	34.4	8.4	6.2	1	7	4	5	5	1	3
		(6.9)	(11.2)	(8.1)	(2.4)	(10.0)	(70.0)	(40.0)	(50.0)	(50.0)	(10.0)	(30.0)
Not satisfied	8	33.5	28.5	6.0	6.4	0	5	8	5	5	2	6
		(9.7)	(3.9)	(5.0)	(2.2)	(0.0)	(62.5)	(100.0)	(62.5)	(62.5)	(25.0)	(75.0)

Saudi Airlines Ticket reservation operators												
Very satisfied	35	76.2	39.6	18.9	6.0	22	7	7	7	7	5	7
		(14.4)	(8.4)	(11.5)	(0.0)	(62.9)	(20.0)	(20.0)	(20.0)	(20.0)	(14.3)	(20.0)
Satisfied	37	66.8	38.9	19.4	6.0	11	16	18	14	8	11	17
		(11.6)	(10.5)	(11.3)	(0.0)	(29.7)	(43.2)	(48.6)	(37.8)	(21.6)	(29.7)	(45.9)
Satisfaction to some extent	14	74.0	37.8	17.6	6.0	4	7	6	8	2	3	3
		(11.0)	(8.0)	(9.1)	(0.0)	(28.6)	(50.0)	(42.9)	(57.1)	(14.3)	(21.4)	(21.4)
Not satisfied	14	68.6	40.8	19.3	6.0	4	10	8	6	5	4	7
		(11.6)	(7.6)	(8.6)	(0.0)	(28.6)	(71.4)	(57.1)	(42.9)	(35.7)	(28.6)	(50.0)

Saudi Telecom Co. computer operators												
Very satisfied	29	82.1	30.1	7.2	7.5	6	14	11	16	7	11	4
		(16.6)	(7.3)	(7.2)	(2.1)	(20.9)	(48.3)	(37.9)	(55.2)	(24.1)	(37.9)	(13.8)
Satisfied	31	84.1	31.0	7.5	6.9	5	22	21	15	8	16	6
		(11.0)	(8.7)	(7.4)	(2.0)	(16.1)	(71.0)	(67.7)	(48.4)	(25.8)	(51.6)	(19.4)
Satisfaction to some extent	27	79.3	28.5	5.1	7.0	5	17	18	13	7	14	2
		(14.8)	(5.6)	(6.3)	(2.2)	(18.5)	(63.0)	(66.7)	(48.1)	(25.9)	(51.9)	(7.4)
Not satisfied	13	78.4	29.0	7.3	7.0	0	10	9	7	4	9	1
		(15.1)	(6.2)	(7.5)	(2.2)	(0.0)	(76.9)	(69.2)	(53.8)	(30.8)	(69.2)	(7.7)

**Table 16:** Incidence of eye and vision complaints as related to previous ailments of computer users/operators.

Complaints	Number of operators	Ergonomic score mean ± SD	Age (year) mean ± SD	Duration of employment (year) mean ± SD	Computer use (hours/day) mean ± SD	Complaints *N* (%)		
						None	General	Eye and vision
King Abdulaziz University computer users								
None	** 58**	36.6	30.9	5.4	6.0	12	27	25
		(7.5)	(9.2)	(5.6)	(2.0)	(20.7)	(46.6)	(43.1)
Short-sighted	** 30**	37.6	31.3	5.4	6.6	1	20	25
		(8.3)	(8.8)	(5.6)	(3.2)	(3.3)	(66.7)	(83.3)
Long-sighted	** 7**	37.2	40.4	18.0	5.7	1	3	3
		(6.7)	(11.4)	(10.8)	(2.0)	(14.3)	(42.9)	(42.9)
Others	** 7**	36.5	39.8	11.9	5.6	1	2	5
		(4.9)	(13.7)	(12.3)	(2.1)	(14.3)	(28.6)	(71.4)

Saudi Airlines Ticket reservation operators								
None	** 70**	71.4	38.9	18.5	6.0	41	18	13
		(13.0)	(9.1)	(10.9)	(0.0)	(58.6)	(25.7)	(18.6)
Short-sighted	** 23**	72.2	38.7	18.9	6.0	0	17	19
		(13.2)	(10.6)	(11.4)	(0.0)	(0.0)	(73.9)	(82.6)
Long-sighted	** 2**	65.0	47.5	26.5	6.0	0	2	2
		(12.8)	(0.7)	(0.7)	(0.0)	(0.0)	(100.0)	(100.0)
Others	** 5**	68.8	43.6	22.8	6.0	0	3	5
		(19.0)	(4.8)	(6.1)	(0.0)	(0.0)	(60.0)	(100.0)

Saudi Telecom Co. computer operators								
None	** 58**	80.4	29.1	5.8	7.2	13	29	24
		(14.9)	(6.9)	(6.0)	(2.0)	(22.4)	(50.0)	(41.4)
Short-sighted	** 24**	84.8	30.3	8.1	6.7	3	19	20
		(12.9)	(6.2)	(7.6)	(2.1)	(12.5)	(79.2)	(83.3)
Long-sighted	** 11**	80.0	30.5	6.6	7.2	0	8	8
		(13.5)	(9.5)	(8.4)	(2.1)	(0.0)	(72.7)	(72.7)
Others	** 7**	86.0	32.7	10.0	7.4	0	7	7
		(14.6)	(9.3)	(10.3)	(2.7)	(0.0)	(100.0)	(100.0)

**Table 17:** Incidence of musculoskeletal complaints as related to previous ailments of computer users/operators.

Complaints	Number of operators	Ergonomic score mean ± SD	Age (year) mean ± SD	Duration of employment (year) mean ± SD	Computer use (hours/day) mean ± SD	Complaints *N* (%)					
						None	General	Neck and shoulder	Upper extremity	Lower extremity	Trunk
King Abdulaziz University computer users											
None	** 59**	37.6	31.6	6.5	6.0	13	24	20	17	6	20
		(7.7)	(9.0)	(6.6)	(2.4)	(22.0)	(40.7)	(33.9)	(28.8)	(10.2)	(33.9)
Neck	** 22**	36.0	29.7	6.3	5.9	1	13	21	9	3	15
		(7.3)	(8.2)	(6.6)	(1.5)	(4.5)	(59.1)	(95.5)	(40.9)	(13.6)	(68.2)
Shoulder and arms	** 11**	37.2	29.5	7.2	6.4	1	9	9	8	2	7
		(8.8)	(8.8)	(7.8)	(1.5)	(9.1)	(81.8)	(81.8)	(72.7)	(18.2)	(63.6)
Lower trunk	** 13**	32.2	31.4	7.8	7.4	0	11	11	3	3	12
		(8.7)	(8.5)	(7.3)	(3.6)	(0.0)	(84.6)	(84.6)	(23.1)	(23.1)	(92.3)
Thigh and leg	** 5**	33.4	34.8	9.6	8.2	0	4	2	1	3	3
		(7.0)	(15.8)	(9.0)	(5.6)	(0.0)	(80.0)	(40.0)	(20.0)	(60.0)	(60.0)
Others	** 1**	41.0	35.0	2.0	7.0	0	1	0	0	0	1
		(0.0)	(0.0)	(0.0)	(0.0)	(0.0)	(100.0)	(0.0)	(0.0)	(0.0)	(100.0)

Saudi Airlines Ticket reservation operators											
None	** 62**	71.4	38.4	18.7	6.0	39	11	8	4	4	7
		(13.6)	(9.1)	(11.2)	(0.0)	(62.9)	(17.7)	(12.9)	(6.5)	(6.5)	(11.3)
Neck	** 24**	74.4	39.8	19.4	6.0	0	24	21	12	11	16
		(13.4)	(8.9)	(9.7)	(0.0)	(0.0)	(100.0)	(87.5)	(50.0)	(45.8)	(66.7)
Shoulder and arms	** 11**	75.4	42.3	21.8	6.0	0	10	11	7	5	7
		(10.4)	(8.4)	(10.0)	(0.0)	(0.0)	(90.9)	(100.0)	(63.6)	(45.5)	(63.6)
Lower trunk	** 23**	70.6	41.7	22.4	6.0	1	15	15	12	14	18
		(13.6)	(10.1)	(10.4)	(0.0)	(4.3)	(65.2)	(65.2)	(52.2)	(60.9)	(78.3)
Thigh and leg	** 8**	73.4	40.2	19.0	6.0	1	7	5	6	5	6
		(15.2)	(7.7)	(8.9)	(0.0)	(12.5)	(87.5)	(62.5)	(75.0)	(62.5)	(75.0)
Others	** 1**	78.0	45.0	20.0	6.0	0	1	1	0	1	1
		(0.0)	(0.0)	(0.0)	(0.0)	(0.0)	(100.0)	(100.0)	(0.0)	(100.0)	(100.0)

Saudi Telecom Co. computer operators											
None	** 55**	81.1	29.4	6.4	7.3	13	31	24	7	20	7
		(14.5)	(7.7)	(6.9)	(2.0)	(23.6)	(56.4)	(43.6)	(12.7)	(36.4)	(12.7)
Neck	** 17**	82.9	30.1	7.9	6.9	0	16	13	8	12	3
		(11.8)	(5.8)	(7.9)	(1.5)	(0.0)	(94.1)	(76.5)	(47.1)	(70.6)	(17.6)
Shoulder and arms	** 4**	84.0	30.0	7.5	6.5	0	4	2	0	1	0
		(9.3)	(4.6)	(7.3)	(1.0)	(0.0)	(100.0)	(50.0)	(0.0)	(25.0)	(0.0)
Lower trunk	** 16**	82.3	32.3	8.6	6.8	1	10	7	6	12	1
		(15.1)	(8.5)	(8.4)	(3.0)	(6.3)	(62.5)	(43.8)	(37.5)	(75.0)	(6.3)
Thigh and leg	** 4**	84.5	27.2	4.6	7.5	0	1	2	3	3	1
		(12.0)	(3.9)	(4.2)	(1.9)	(0.0)	(25.0)	(50.0)	(75.0)	(75.0)	(25.0)
Others	** 4**	72.0	27.7	3.0	6.3	2	1	3	2	2	1
		(25.9)	(3.5)	(2.1)	(2.8)	(50.0)	(25.0)	(75.0)	(50.0)	(50.0)	(25.0)

**Table 18:** Freedom of computer users/operators from complaints as related to workstation score number (percent).

Score of workstation	KAU computer users				Saudi Airlines Ticket reservation operators				Saudi Telecom Co. computer operators			
	Operator sample	No general complaints	No eye and vision complaints	No musculo-skeletal complaints	Operator sample	No general complaints	No eye and vision complaints	No musculo-skeletal complaints	Operator sample	No general complaints	No eye and vision complaints	No musculo-skeletal complaints
<50	8	3	3	1	—	—	—	—	3	1	1	0
		(37.5)	(37.5)	(12.5)						(33.3)	(33.3)	(0.0)
50–59	10	4	3	6	21	11	11	13	3	2	2	2
		(40)	(30)	(60)		(52.4)	(52.4)	(61.9)		(66.7)	(66.7)	(66.7)
60–69	15	8	6	7	22	13	14	14	11	3	3	3
		(53.3)	(40)	(46.7)		(59.1)	(63.6)	(63.6)		(27.3)	(27.3)	(27.3)
70–79	26	13	15	13	35	24	23	18	24	8	13	12
		(50)	(57.7)	(50)		(68.6)	(65.7)	(51.4)		(33.3)	(54.2)	(50)
80–89	25	12	10	10	8	4	3	4	23	11	11	12
		(48)	(40)	(40)		(50)	(37.5)	(50)		(47.8)	(47.8)	(52.2)
90–100	16	9	5	9	14	8	10	9	36	12	11	15
		(56.3)	(31.3)	(56.3)		(57.1)	(71.4)	(64.3)		(33.3)	(30.6)	(41.7)
Total	**100**	** **	** **	** **	**100**	** **	** **	** **	**100**	** **	** **	** **
